# Dupuytren's Treatment of Scrofula

**Published:** 1830-04-01

**Authors:** 


### Dupuytren's Treatment of Scrofula.

The following note respecting the above celebrated surgeon's method of treatment in scrophulous affections, was communicated by Professor Guilbert to M. Ratier, for the third edition of his work on the Parisian Hospitals, recently translated by Dr. M'Lellan.

"The treatment employed by M. Dupuytren in scrofula differs much from the methods of treatment generally followed, and is the result of observations, anatomical and physiological, on the nature and progress of that disease.

"Whatever be its varieties or its seat, scrofula exhibits three distinct periods in its march. In the first, the disease is in some measure inert, manifesting itself only through the characters proper to the lymphatic constitution, and by an interruption, more or less difficult to perceive, in the action of the parts affected. In this first period, M. Dupuytren employs all the means afforded by the *hygiene* suited to fortify the constitution, and, by consequence, effect the resolution of the disease. He is careful, moreover, to avoid every thing that might irritate, agitate, or heat, as elixirs, antiscorbutic syrups, and other spirituous medicines, which he believes are calculated to make the disease pass from the inert into the inflammatory state.

"It is especially in the second state of the disease, marked always by excitement, fever, local pains, swelling, and sanguineous exhalations, that he sedulously shuns those stimulating remedies which, from the abuse made of them for many years, have produced more evil than the disease itself they were professed to ameliorate.

"In this second period of the malady, M. Dupuytren, without regard to its supposed nature, treats it as an inflammatory affection, by bleeding, leeches, and diet, and by so doing has often arrested its progress, and prevented its melancholy consequences, such as caries of the bones, gibbosities, spontaneous luxations, suppuration, and destruction of the organs. If suppuration be established, and its products escape easily by an external outlet, and if the disease have returned to that almost inert state which constitutes its first period, he resumes the use of the means calculated to strengthen the system, but is still careful to reject every thing that would excite or have a tendency to cause insomnia or fever. For the same reason, he abstains, in the third period of the disease, from the use of vinous, alcoholic, or alkaline preparations As a substitute for such, he prescribes only the purely aqueous preparations of cinchona, gentian, or simarouba ; persuaded that they contain all that is really *tonic* in these substances, and are free from the irritating properties, contained both in the base and vehicle of the ordinary remedies. He thus employs the aqueous infusions, and syrups of gentian, cinchona, and simarouba, to which he gives more or less strength, according to the age and sex of the individual, or the seat and character of the affection."

